# MAIT cells altered phenotype and cytotoxicity in lupus patients are linked to renal disease severity and outcome

**DOI:** 10.3389/fimmu.2023.1205405

**Published:** 2023-10-10

**Authors:** Elena Litvinova, Carine Bounaix, Guillaume Hanouna, Jennifer Da Silva, Laura Noailles, Lucie Beaudoin, Michael Padden, Nessrine Bellamri, Agnès Lehuen, Eric Daugas, Renato C. Monteiro, Héloïse Flament

**Affiliations:** ^1^ Service d’Immunologie, Hôpital Bichat-Claude Bernard, Paris, France; ^2^ Université Paris Cité, Centre de Recherche sur l’Inflammation Institut national de la santé et de la recherche médicale (INSERM) Unité Mixte de Recherche (UMR)1149 & Centre national de la recherche scientifique (CNRS) équipe mixte de recherche (EMR)8252, Inflamex Laboratory of Excellence, Paris, France; ^3^ Service de Néphrologie, Assistance publique - Hôpitaux de Paris (AP-HP), Hôpital Bichat-Claude Bernard, Paris, France; ^4^ Université de Paris, Institut Cochin, Institut national de la santé et de la recherche médicale (INSERM) U1016, Centre national de la recherche scientifique (CNRS) Unité Mixte de Recherche (UMR) 8104, Inflamex Laboratory of Excellence, Paris, France

**Keywords:** MAIT cell, lupus nephritis, granzyme B, activation, prognosis markers

## Abstract

**Introduction:**

Systemic lupus erythematosus (SLE) is an autoimmune disease in which circulating immune complexes can cause different types of glomerulonephritis, according to immune deposits and to the type of glomerular cell injury. Proliferative lesions represent the most severe form of lupus nephritis (LN) and often lead to kidney failure and death. Mucosal-associated invariant T (MAIT) cells are a subset of innate-like T cells that recognize microbial-derived ligands from the riboflavin synthesis pathway. Although abundant in peripheral blood, MAIT cells are enriched in mucosal and inflamed tissues. While previous studies have reported concordant results concerning lower MAIT cell frequencies in the blood of SLE patients, no information is known about MAIT cell function and LN severity and outcome.

**Methods:**

In the current study, we analyzed the baseline phenotype and function of peripheral blood MAIT cells by flow cytometry in 26 patients with LN and in a control group of 16 healthy individuals.

**Results:**

We observe that MAIT cell frequencies are markedly reduced in blood of LN patients. MAIT cells from patients have an altered phenotype in terms of migration, proliferation and differentiation markers, notably in most severe forms of LN. Frequencies of PMA/ionomycin stimulated MAIT cells secreting effector molecules, such as proinflammatory IL-17 and cytotoxic protein granzyme B, are higher in LN patients. Patients undergoing a complete renal remission after immunosuppressive therapy had higher MAIT cell frequency, lower expression of proliferation marker Ki-67 and granzyme B (GzB) at inclusion. Remarkably, GzB production defines a predictive model for complete remission.

**Discussion:**

We report here that blood MAIT cells display proinflammatory and cytotoxic function in severe lupus nephritis which may play a pathogenesis role, but without association with systemic lupus activity. Finally, low cytotoxic profile of MAIT cells may represent a promising prognostic factor of lupus nephritis remission one year after induction therapy.

## 1 Introduction

The hallmark of systemic lupus erythematosus (SLE), a systemic autoimmune disease, is the presence of pathogenic autoantibodies, including high titers of double-stranded anti-DNA antibodies (anti-dsDNA). Tissue deposition of autoantigen-autoantibody complexes (immune complexes) induces immune activation and causes inflammatory damage that affects various types of organs, mainly joints, skin, kidneys, and nervous system. About half of SLE patients develop kidney disease. The pathogenesis of lupus nephritis (LN) involves immune complexes renal deposition in addition to other injuries resulting in endothelial, podocytic, and tubulointerstitial lesions (1). LN displays diverse clinical presentations and outcomes. Multiple parameters are associated with complications and prognosis of LN, among them socio-demographic and economic factors, but also histopathological features and serological markers. In SLE patients, LN is linked to higher morbidity, characterized by evolution to chronic glomerular lesions leading to end stage renal disease (ESRD) requiring replacement therapy. Furthermore, LN remains the leading cause of mortality in SLE patients (2–5).

Therapeutic care of LN is mainly decided according to histopathologic characteristics determined on the renal biopsy at diagnosis. LN classification is established according to the type of glomerular injuries characterized by activity and chronicity indices. Whereas class I and II generally do not require any specific treatment, class III and IV proliferative LN, the most severe forms of the disease, require an early diagnostic in order to setup immunosuppressive treatment as soon as possible. Treatment of class V remains debated. Despite immunosuppressive medication guidelines (6, 7), about 15% of patients progress to ESRD within 10 years after initial diagnosis. Furthermore, 40% of SLE patients with class III or class V LN will evolve into chronic kidney disease (8). For the management of the most severe forms of LN, induction therapy is prescribed to initiate remission, while maintenance therapy is implemented to prevent relapses.

To date, there is no reliable biomarker to predict LN remission at initiation of induction therapy, and how to distinguish patients in whom remission will be obtained with standard therapy, from patients who will require more intensive induction therapy (9–11). Despite being routinely performed to perform histopathologic classification of LN, kidney biopsy is not a sufficient diagnosis tool as it fails to predict the renal response to standard immunosuppressive therapy. Renal biopsy is also a costly and invasive procedure with potential risks that cannot be repeated in the monitoring of LN. Current biomarkers, that are conventionally used for LN follow-up like albuminuria, urine protein/creatinine ratio (UPCR), estimated glomerular filtration rate (eGFR), anti-dsDNA levels, and serum complement fractions have not been very effective prognostic tools in predicting response to induction immunosuppressive therapy and estimating the probability of renal relapse (1).

Innate-like T cells are characterized as a cell subset that differs from conventional T cells by expressing a restricted T-cell receptor (TCRs) repertoire. Among them, Mucosal-associated invariant T (MAIT) cells are relatively abundant in the liver, at mucosal sites and in the blood, where they represent 1-10% of whole T cell population in humans. MAIT cells express a semi-invariant TCR restricted to a non-polymorphic MHC-related molecule, called MR1. Human MAIT cells express the TCRVα7.2-Jα33 chain associated with a limited number of TCRβ chains (12). MAIT cells recognize non-peptide antigens including bacteria and fungi-derived riboflavin and folate (vitamins B2 and B9) precursors and byproducts (13, 14). MAIT cells highly express many NK-cell specific markers such as the C-type lectin receptor CD161, in which co-expression with TCRVα7.2 classically defines human MAIT cells (15). MAIT cells also express inflammatory cytokine receptors such as IL-12R and IL-18Rα at steady state (16). Upon activation through TCR signaling (17), or cytokine stimulation (16), MAIT cells are able to respond as effector cells and are involved in mucosal immunity, but also in pathologic inflammation. This activation can induce release of Th1/Th17 cytokines, such as IFN-γ, TNF-α, and IL-17 as well as apoptosis induction by perforin and cytotoxic molecule granzyme B (GzB). MAIT cells may therefore fulfill important key functions in the early defense against microbial pathogens at mucosal barriers (18). In addition to their antimicrobial functions, MAIT cells could also participate in local immunity homeostasis by preserving epithelial and mucosal barrier integrity. Indeed, loss of MAIT cells during infectious diseases like HIV infection (19) or autoimmune diseases like type 1 diabetes (20) may dampen intestinal barrier integrity promoting microbial translocation with deleterious effects on inflammation. Through their ability to respond to inflammatory cytokines, MAIT cells may also be implicated in the pathogenesis of chronic inflammatory diseases, autoimmune or metabolic diseases (21, 22). Increased quantities of MAIT cells were detected in tissue lesions in mouse models and/or patients with type 1 diabetes (20), inflammatory bowel disease (23), obesity (24), psoriasis (25), multiple sclerosis (26), and rheumatoid arthritis (27).

In SLE patients, circulating MAIT cells frequencies were found to be lower than in controls (27, 28). However, frequency of peripheral apoptotic MAIT cells was increased and remaining circulating cells had an impaired phenotype expressing the activation marker CD69 (28) and the activation/exhaustion marker PD1, as well as displaying an impaired Ca^2+^/calcineurin/NFAT1 signaling pathway (27) that leads to be less responsive to stimuli. These studies suggested that MAIT cells could be lost in SLE due to activation-induced apoptosis *in vivo*.

Although it was suggested that MAIT cell activation positively correlates with disease activity in SLE (28, 29), their role in LN remained undetermined. Here, we aimed to define whether circulating MAIT cells could offer a diagnostic value as biomarkers of renal disease activity as well as a prognostic value following therapeutic approaches in active LN.

## 2 Materials and methods

### 2.1 Participants and clinical data

Patients with LN, and healthy individuals who did not report history of neither SLE nor inflammatory diseases, were recruited in Bichat Hospital between November 2017 and Mai 2019. Patients aged over 18-years old and presenting with a biopsy-proven LN were included in the study. Patients presented four or more criteria of the 1982 revised American College of Rheumatology (ACR) SLE classification. They did not have history of renal transplantation, surgery, cancer, urinary tract infection and systemic infection. Healthy individuals were also included as controls.

At diagnostic, demographic and laboratory data were collected: age, sex, serum albumin, blood creatinine, urine creatinine and 24-hour proteinuria. Systemic lupus activity was estimated according to the SLE Disease Activity Index (SELENA SLEDAI) (30), a SLEDAI score ≥ 5 defining active SLE. LN class as well as activity and chronicity indexes according to the ISN/RPS 2003 classification were also collected. Class III and class IV LN with active lesions on kidney biopsy were defined as active class III or active class IV LN, respectively. Conversely, a non-active class III or class IV LN was defined by the absence of activity lesions on kidney biopsy. In addition, class I, class II and class V were classified as non-active LN.

The criteria for remission or absence of remission at 12 months were defined as follows, using eGFR and UPCR. Complete remission was considered when UPCR < 0.5 g/g and eGFR ≥ 60 ml/min/1.73m^2^, or if < 60 ml/min/1.73m^2^ at baseline, and no decline (>20%) as compared to baseline. Partial renal response was defined as 50% improvement in UPCR compared to baseline and UPCR between 0.5 and 3 g/g and eGFR ≥ 60 ml/min/1.73m^2^, or if < 60 ml/min/1.73m^2^ at baseline, no decline >20% compared to baseline. The absence of remission was defined as absence of complete or partial remission.

### 2.2 Cell isolation and activation

Peripheral blood mononuclear cells (PBMCs) isolation was performed on heparinized whole blood using Lymphosep (Biosera) according to the manufacturer’s instructions. Production of cytokines was measured after *in vitro* TCR-independent stimulation for 6 hours at 37°C. The latter was performed in RPMI medium (Gibco) supplemented with 10% fetal bovine serum (Gibco) with PMA at 25 ng/ml and ionomycin at 1 μg/ml, in the presence of brefeldin A at 10 μg/ml (all from Sigma Aldrich).

### 2.3 Flow cytometric analysis

The list of Monoclonal antibodies (mAb) used in this study is presented in **Figure S1**. For cell surface staining, PBMC were incubated with appropriate mAb in PBS with 1% BSA (FACS buffer) for 30 min on ice, followed by washes in FACS buffer prior to acquisition or subsequent intracellular or intranuclear staining. For intracellular staining, a fixation/permeabilization solution kit was used (Cytofix/Cytoperm, BD). For intranuclear staining, Foxp3/transcription factor staining buffer set was used (eBiosciences). Data was acquired using a BD Biosciences LSRFortessa cytometer, and analyzed by FlowJo software (Tree Star). Gating strategy for MAIT cell identification is shown in **Figure S2**. Briefly, dead cells were excluded by stringent FSC/SSC gating, and doublets were excluded by SSC-H/SSC-A and FSC-H/FSC-A gating. Then T cells were gated as CD3^+^ cells, and γδT cells were removed by negative gating as well as iNKT cells (TCRVa24+) and CD4 T cells. Finally, MAIT cells were identified as TCRVα7.2^+^ and CD161^high^ cells, which can be either CD8^+^ or double negative CD4^-^CD8^-^. The experiments (PBMC isolation, flow cytometry phenotyping on fresh cells and after PMA/ionomycin restimulation *in vitro*) were performed only once for each patient and healthy control. Some flow cytometry experiments were not performed for all parameters due to limited number of cells. Representative flow images and gating strategies used to analyze expression of CD25, CD69, CD27, KLRG1, Ki-67, CD127, CD56 and CCR6 markers on MAIT cells are shown in **Figure S3**. **Figure S4** shows representative flow images and gating strategies used to analyze intracellular expression of IL-2, IL-4, IL-10, IL-17α, IFN-γ, TNF-α and granzyme B (GzB) on MAIT cells following PBMC stimulation with PMA/ionomycin for 6 hours.

### 2.4 Statistical analyses

Mann-Whitney U or Unpaired t-test, when appropriate, were used for nonparametric tests. Spearman’s correlation test was used to perform Correlation analyses. Two-sided P values less than 0.05 were considered statistically significant. Statistical analyses were performed using GraphPad software (GraphPad Prism).

### 2.5 Study approval and ethical statement

The study was approved by the “comité de protection des personnes” (Paris, France) under reference ID-RCB-2014-A00809-38. All patients provided a written informed consent.

## 3 Results

### 3.1 Patients

**Figure 1:**
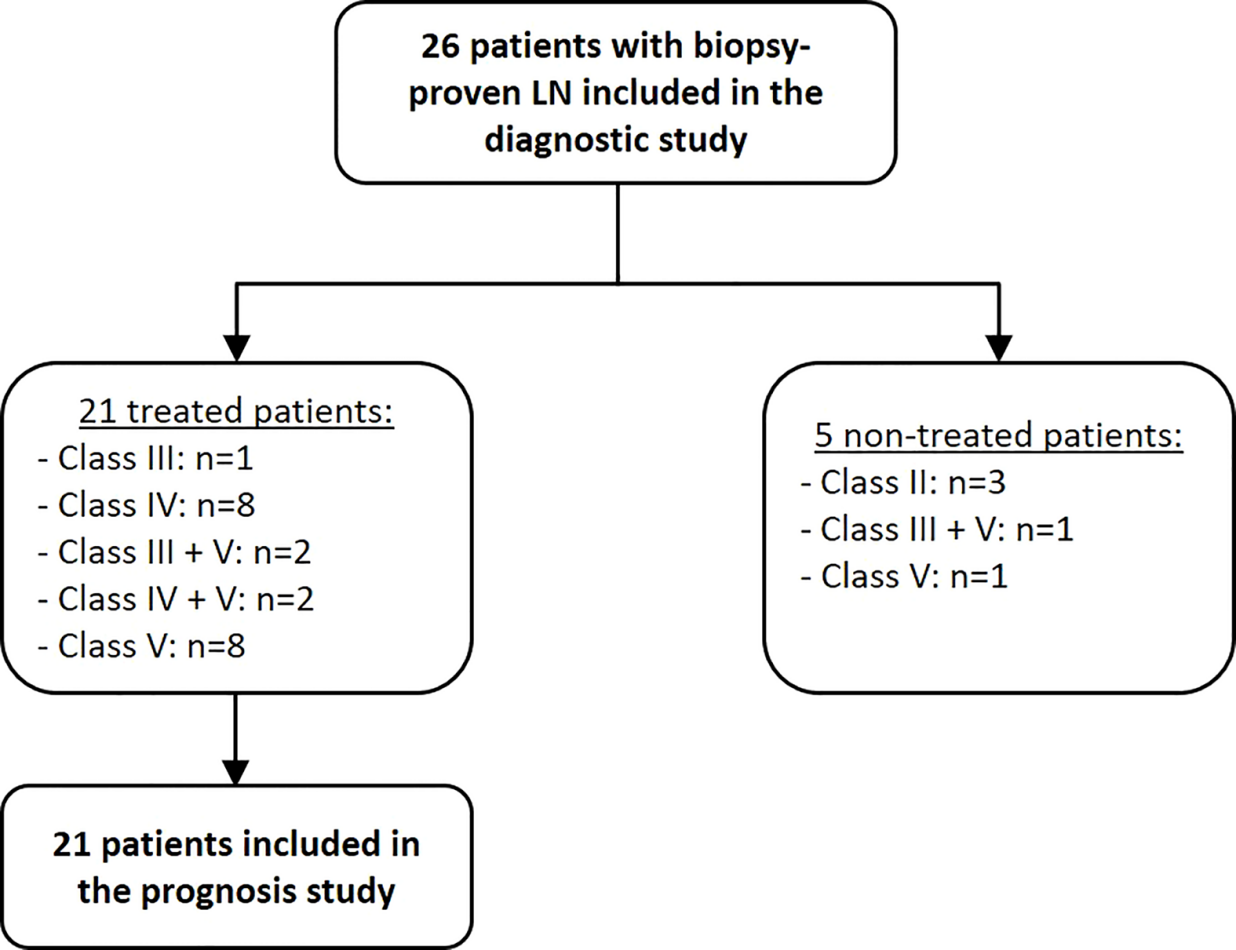
Flow chart of the 26 enrolled LN patients.

All characteristics of studied population are shown in **Figure 1** and **Table 1** including 26 LN patients with a mean age of 32 ± 11 years (88% were female). 13 patients (50%) were diagnosed as having an active LN (class III/IV +/-V). 21 patients were treated with an induction therapy in which 13 of them have either class III or a class IV +/-V LN with activity. 8 additional patients have pure LN class V with nephrotic syndrome. After 12 months of induction therapy, 12 patients (57%) achieved complete response, 3 (14%) partial response and the other 6 (29%) were nonresponsive (**Table 2**). 16 healthy volunteers (mean age of 42 ± 14 years) were included into the control group. 10 healthy controls (63%) were women.

**Table 1:** Demographic, clinical and laboratory data of LN patients and healthy controls.

	HC (n = 16)	LN (n = 26)	
Variables	N (%) or mean ± SD	N (%) or mean ± SD	Median with IQR
Female	10 (63)	23 (88)	–
Age (years)	42 ± 14	32 ± 11	28 [24, 38]
Baseline SLEDAI score	–	12 ± 7	10 [8, 15]
Baseline SLEDAI score ≥ 5	–	23 (88)	–
ISN/RPS class			
Class II	–	3 (12)	–
Class III	–	1 (4)	–
Class IV	–	8 (31)	–
Class III + V	–	3 (12)	–
Class IV + V	–	2 (8)	–
Class V	–	9 (35)	–
IS agent at inclusion	–	8 (31)	–
Laboratory data			
Serum albumin (g/dL)	–	2.9 ± 0,7	30 [25, 34]
Estimated (e) GFR (mL/min/1.73 m^2^)	–	80 ± 19	87 [73, 90]
UPCR (g/g creatinine)	–	2.8 ± 1.9	2.2 [1.1, 4.3]
		Active LN (n = 13)	
Renal activity index	–	52 ± 23	58 [35, 70]
Renal chronicity index	–	25 ± 18	30 [15, 32]

LN, lupus nephritis; SD, standard deviation; IQR, Interquartile Range, SLEDAI, Systemic Lupus Erythematosus Disease Activity Index; ISN/RPS, International Society of Nephrology/Renal Pathology Society; GFR, Glomerular Filtration Rate; UPCR, Urine Protein Creatinine Ratio; IS, Immunosuppressive.

**Table 2:** Conventional biomarkers of renal response.

	Complete response	Partial response	Non-response	p-value
N (%)	12 (57)	3 (14)	6 (29)	–
Age	27 [24, 30]	35 [31, 39]		0.07
Baseline SLEDAI score	11 [9, 18]	9 [7, 13]		0.14
Renal activity index*	56 [28, 69]	66 [56, 70]		0.61
Renal chronicity index*	23 [8, 34]	30 [26, 31]		0.73
Laboratory data				
Serum albumin (g/dL)	29 [23, 34]	28 [25, 30]		0.87
Estimated (e) GFR (mL/min/1,73 m^2^)	90 [74, 90]	82 [73, 90]		0.97
UPCR (g/g creatinine)	2.0 [1.3, 3.6]	2.5 [1.3, 4.1]		0.49

Complete renal response: UPCR < 0.5 g/g and eGFR ≥ 60 ml/min, or if < 60 ml/min at baseline with no decline >20% compared to baseline; Partial renal response: 50% improvement in UPCR and UPCR between 0.5 and 3 g/g and stabilization (< 20% decrease) of eGFR. Values are given as median [interquartile range]. *Complete response vs. partial- or non-response among active LN only (n = 13, class III or IV ± V), Mann-Whitney test.

### 3.2 Conventional biomarkers are not related to renal response

As shown in **Table 2**, conventional biomarkers such as age, sex, renal SLE Disease Activity Index (SLEDAI) score, renal activity/chronicity indexes, serum albumin level, eGFR or UPCR were not significantly different between patients with complete, partial or no response after induction therapy.

### 3.3 Circulating MAIT cell frequencies are markedly reduced in patients with lupus nephritis

**Figure 2:**
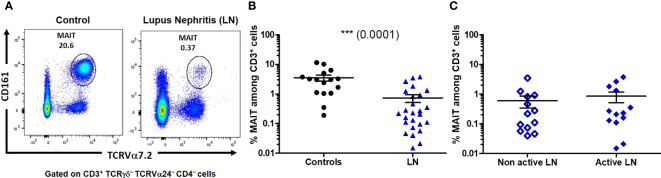
The proportion of mucosal-associated invariant T (MAIT) cells is decreased among lupus nephritis (LN) patients. **(A)** Representative profile of MAIT cells, defined as CD161^high^ TCRVα7.2^+^, gated on CD3^+^ TCRγδ^-^ TCRVα24^-^ CD4^-^ cells. **(B)** Percentage of MAIT cells among T cells (CD3^+^) in controls (*n* = 16) and LN (*n* = 26). **(C)** Percentage of MAIT cells among T cells (CD3^+^) in active (III or IV +/-V, *n* = 13) and non-active (II and pure class V, *n* = 13) LN. Mann-Whitney U-test, horizontal lines are mean ± SD values.

Frequency of circulating MAIT cells (identified as CD3^+^ TCRγδ^−^ TCRVα24^−^ CD4^-^ CD161^high^ Vα7.2^+^ cells that were either CD8^+^ or double negative CD4^-^CD8^-^) was first evaluated in the PBMC from patients and healthy controls (**Figure 2A**). The median MAIT cell frequency (defined as the percentage of MAIT cells among total T-cell population) was strongly decreased in LN patients as compared to controls (*p* = 0.0001). While 3.06% (0.19-11.5) of MAIT cells was observed in controls, within the range reported by others (16, 19, 23), only 0.25% (0.02-3.76) was seen in LN patients (**Figure 2B**). However, no significant difference in MAIT cell frequency was seen within different type of LN patients neither according to histologic classification (not shown) nor according to active (III or IV +/-V) and non-active (II and pure class V) status (**Figure 2C**).

### 3.4 Increased frequencies of CD127^-^ CCR6^-^ MAIT cells in lupus nephritis patients

**Figure 3:**
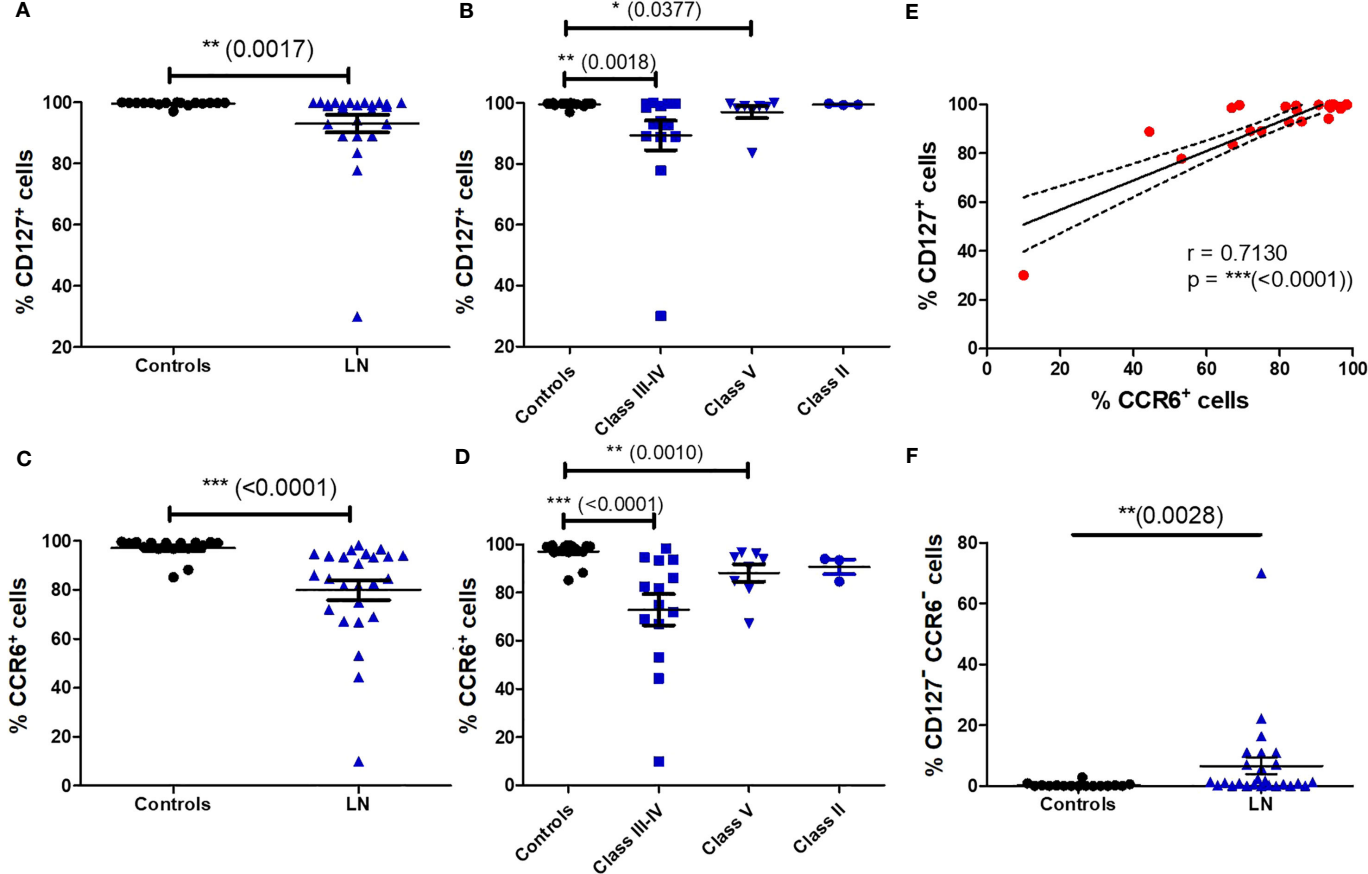
Altered phenotype of blood MAIT cells in LN patients. Percentage of CD127^+^ MAIT cells **(A)** in HC (*n* = 16) vs LN patients (*n* = 26); **(B)** in controls (*n* = 16) vs. LN patients (*n* = 26) classified according to the lupus nephritis WHO classification. Percentage of CCR6^+^ MAIT cells **(C)** in HC (*n* = 16) vs. LN patients (*n* = 26); **(D)** in controls (*n* = 16) vs. LN patients (*n* = 26) classified according to the lupus nephritis WHO classification. Data was analysed with Mann-Whitney U-tests. Horizontal lines are mean ± SD values. **(E)**: correlation of CD127 and CCR6 expression by MAIT cells in LN patients. **(F)**: Percentage of CD127- CCR6- MAIT cells in controls vs. LN patients. * P ≤ 0.05 ; ** P ≤ 0.01 ; *** P ≤ 0.001.

As MAIT cells express tissue-homing chemokine receptors (9), we next evaluated the impact of LN disease activity on the remaining blood MAIT cell population. We found that virtually all blood MAIT cells expressed CD127 (IL-7Rα) and CCR6 (CCL20R) in controls. However, in patients with LN notably classes III and IV, we observed a lower frequency of MAIT cells expressing CD127 (**Figures 3A, B**) and CCR6 (**Figures 3C, D**).

Remarkably, there was also a strong correlation in the expression of CCR6 and CD127 on MAIT cells in LN patients (*p* < 0.0001) (**Figure 3E**). The frequency of double negative CD127^-^ CCR6^-^MAIT cells is increased among LN patients (**Figure 3F**). This phenotype is characteristic of short lived terminally differentiated effector memory T cells, displaying a complete exhaustion phenotype and loss of most cytokines and chemokines receptors (24). However, there was no difference in CD127^-^ or CCR6^-^ MAIT cell frequency between active (III or IV +/-V) and non-active (II and pure class V) LN (**Figure S5**).

**Figure 4:**
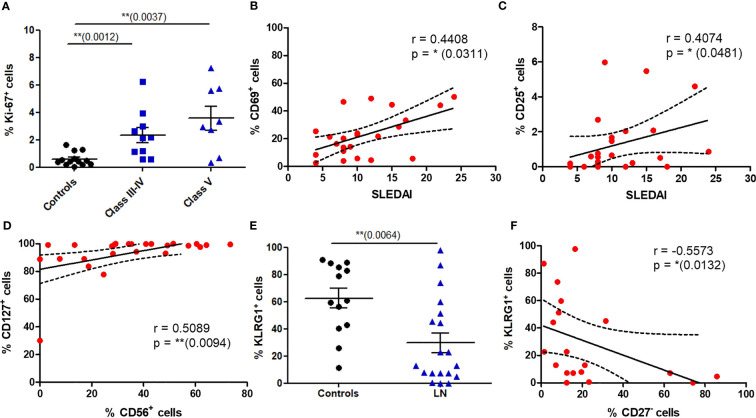
Phenotype of circulating MAIT cells reveals increased proliferative capacity and altered expression of activation markers in LN patients. **(A)** Proportion of MAIT cells expressing Ki-67 in controls (*n* = 16) vs. LN patients classified according to the lupus nephritis WHO classification (*n* = 18). **(B, C)** Correlation between the SLEDAI score and the percentage of MAIT cell expressing CD69 and CD25 in LN patients (*n* = 26). **(D)** Correlation of CD127 and CD56 expression by MAIT cells in LN patients (*n* = 26). **(E)** Frequency of MAIT cells expressing KLRG1 in controls (*n* = 13); vs. LN patients (*n* = 19). **(F)** Correlation between expression of KLRG1 and CD27 by MAIT cells in LN patients (*n* = 19). Data was analysed using Mann-Whitney U-tests. Horizontal lines are mean ± SD values. Correlations were analyzed with the Spearman test; dashed line represent the 95% confident interval. * P ≤ 0.05 ; ** P ≤ 0.01.

We next wondered whether circulating MAIT cells from LN patients display proliferation markers using Ki-67 staining. This expression was significantly increased on MAIT cells from patients presenting proliferative LN (class III-IV) and class V (**Figure 4A**). There was no difference in the frequency of Ki-67^+^ MAIT cells between active and non-active LN (**Figure S5**). Despite such proliferation profile, no significant differences were found between LN and controls concerning the expression of activation (CD25 and CD69, not shown) and differentiation (CD27, **Figure S6**) markers. However, there was a positive correlation between the SLEDAI score and the percentage of circulating MAIT cells expressing CD25 (*p* = 0.0481) or CD69 (*p* = 0.0311) (**Figures 4B, C**). We also evaluated the expression of activation/exhaustion marker PD1, which mediate inhibition of TCR-induced activation and proliferation. No differences were found for PD1 levels (**Figure S6**).

### 3.5 Enhanced cytotoxic and Th17 phenotype among MAIT cells in LN

To analyze a putative cytotoxic phenotype of MAIT cells in LN, we first studied the expression of CD56, a Natural Killer (NK) cell marker present on activated and/or cytotoxic MAIT cells. In LN MAIT cells, a positive correlation was indeed observed between the expression of CD56 and IL-7 receptor (CD127) suggesting a cytotoxic phenotype of these cells (**Figure 4D**). Moreover, while most of the MAIT population expressed the NK-associated cytotoxicity marker KLRG1 (Killer-cell Lectin-like Receptor G1) in HC, there was a marked reduction in KLRG1 expression among LN patients (**Figure 4E**). The expression of KLRG1 marker was negatively correlated with CD27^-^ phenotype of MAIT cells in LN (**Figure 4F**). However, we did not find any difference in the frequency of KLRG1^+^ MAIT cells between active and non-active LN (**Figure S5**).

**Figure 5:**
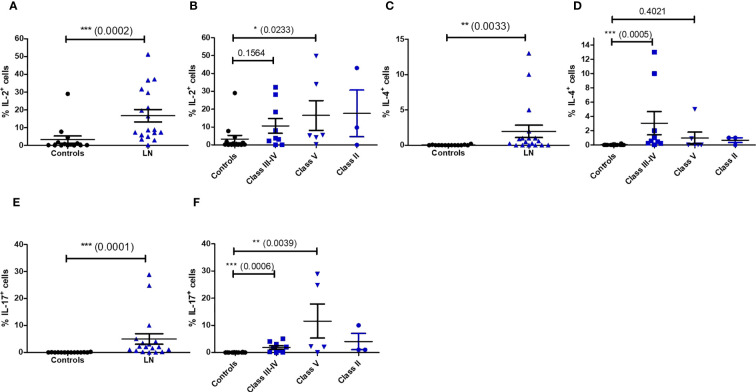
Cytokine production by MAIT cells after PMA/ionomycin restimulation in controls (*n* = 13) and LN patients (*n* = 18) **(A, C, E)**; and according to the lupus nephritis WHO classification **(B, D, F**). Data was analysed with Mann-Whitney U-tests. Horizontal lines are mean ± SD values. * P ≤ 0.05 ; ** P ≤ 0.01 ; *** P ≤ 0.001.

**Figure 6:**
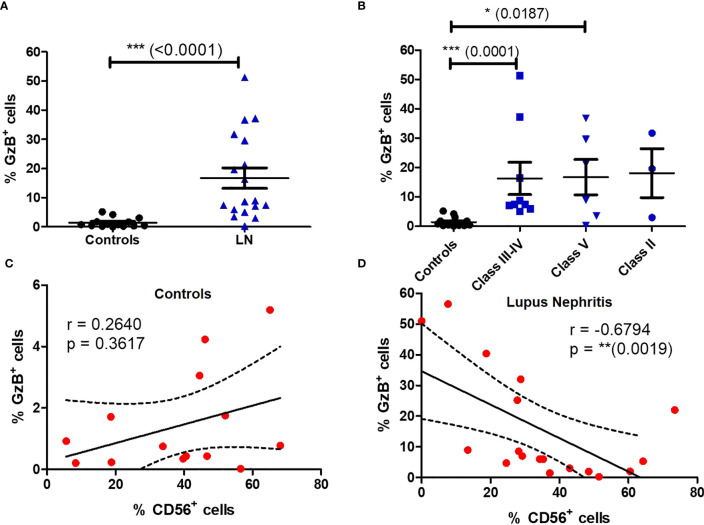
Circulating MAIT cells display a cytotoxic phenotype in LN patients. Frequency of GzB^+^ MAIT cells **(A)** in controls (*n* = 13) vs. LN patients (*n* = 18); **(B)** in controls vs. LN patients classified according to the lupus nephritis WHO classification. Correlation between GzB production and CD56 expression by MAIT cells in LN **(C)** or controls **(D)**. Data were analysed with Mann-Whitney U-tests. Horizontal lines are mean ± SD values. Correlations were analyzed with the Spearman test; dashed line represent the 95% confident interval. * P ≤ 0.05 ; ** P ≤ 0.01 ; *** P ≤ 0.001.

We next wondered if the impaired phenotype of MAIT cells in LN patients was accompanied by functional changes. We therefore activated PBMC with PMA/ionomycin and measured MAIT intracellular production of multiple cytokines such IFN-γ, TNF-α, IL-17, IL-2, IL-10, IL-4 and the cytotoxic effector molecule granzyme B (GzB). Restimulation with PMA and ionomycin, though nonspecific in contrast to antigen stimulation, measures the maximal capacity of lymphocytes to produce cytokines and GzB. While production of IFN-γ, TNF-α ;and IL-10 were not altered between patients and controls (**Figure S6**), significant increase was observed for levels of IL-2, IL-4 and IL-17, especially in classes III-IV (**Figures 5A–F**). However, there was no difference in the frequency of cytokine-producing MAIT cells between active and non-active LN (**Figure S7**). Similarly, increased GzB production was seen in LN-derived MAIT cells as compared to healthy controls (**Figure 6A**). GzB^+^ MAIT cells were mainly present in patients with LN of classes III, IV and V (**Figure 6B**). Unlike other cytotoxicity markers, GzB is only found in activated cytotoxic lymphocytes. Interestingly, in contrast to controls there was a negative correlation between GzB and CD56 expression on MAIT cells from LN patients (**Figures 6C, D**) indicating that circulating GzB^+^ MAIT cells in LN express low levels of CD56. Nevertheless, no difference was noticeable in the frequency of GzB^+^ MAIT cells between active and non-active LN (**Figure S7**).

**Figure 7:**
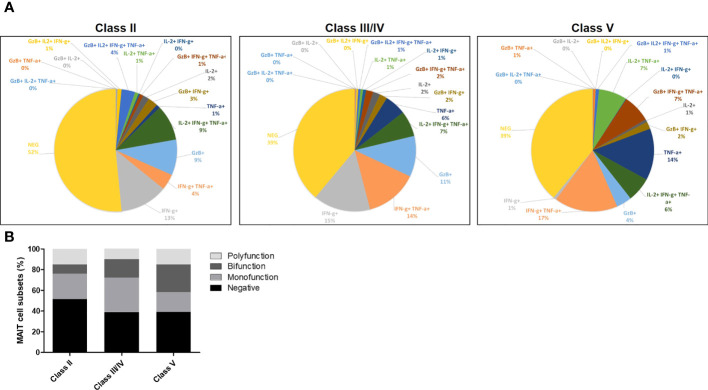
**(A)** Pie charts showing MAIT cells producing cytokines including IL-2, TNF-α, IFN-γ and/or GzB, among LN patients of class II, III, IV and V. **(B)** Bar plot representative of cumulative cytokines production by MAIT cells, classified as producing none (negative), one (monofunction), two (bifunction) or more than two (polyfunction) of these molecules.

Next, we further analyzed the production of major cytokines and GzB by MAIT cells by multivariable analysis (**Figures 7**, **S8**). This analysis showed that MAIT cells secreting GzB (without detection of IL-2, TNF-α, IFN-γ) were increased in patients with LN from class II to class III/IV as compared to LN patients with class V (**Figure 7A**). Moreover, cytokine-producing MAIT cell subsets were classified as producing none, one (monofunctional), two (bifunctional) or more than two (polyfunctional) different cytokines or GzB to display their functional heterogeneity (**Figure 7B**). The results indicate that monofunctional MAIT cells may be more involved in inflammatory LN classes II, III and IV than in class V (**Figure 7B**).

### 3.6 Baseline circulating MAIT cell frequency, Ki-67 expression and granzyme B production are new markers of renal response after induction therapy

**Figure 8:**
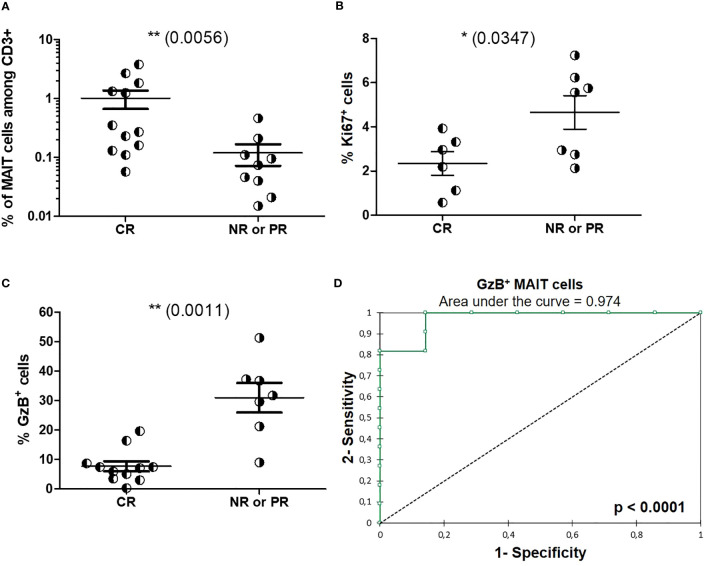
Baseline circulating MAIT cell phenotype as a prognosis marker of one-year renal response following induction therapy. **(A)** Baseline frequency of MAITs cells among CD3^+^ cells in LN patients with complete renal response (RC, *n* = 12) or partial response (PR)/no response (NR) (*n* = 9) at 1 year after induction therapy. **(B)** Baseline frequency of MAITs cells expressing Ki-67 or **(C)** GzB among LN patients with complete renal response (RC) or partial response (PR)/no response) (NR) at 1 year after induction therapy. **(D)** Receiver operating characteristic (ROC) curve of the predictive value of GzB^+^ MAIT cell markers defining the outcome at 1 year after induction therapy. Data were analysed with Mann-Whitney U-tests. Horizontal lines are mean ± SD values. ROC curves were performed with XLSTAT Free software. * P ≤ 0.05 ; ** P ≤ 0.01.

We then assessed baseline circulating MAIT cell frequency and phenotype at time of diagnostic biopsy and before induction treatment initiation, as prognostic factors of the renal response observed one year after induction therapy initiation. Baseline MAIT cell frequencies were higher in LN patients who fulfilled complete response (median 0.31% of T cells) than in patients with partial response or non-response (median 0.08%, **Figure 8A**). Baseline frequency of Ki-67^+^ MAIT cells was significantly lower among LN patients with complete response than among those with partial response or non-response (*p* = 0.0347, **Figure 8B**). There was no correlation between MAIT cell frequency, GzB production, and Ki-67 expression with clinical data such as serum albumin level, UPCR and eGFR (**Figure S9**). Baseline frequency of cytotoxic (GzB^+^) MAIT cells was significantly lower in LN patients who fulfilled complete response than among those with partial response or non-response (*p* = 0.0011, **Figure 8C**). The receiver operating characteristic (ROC) curve analysis was performed to compare the predictive value of blood MAIT cell frequency as well as Ki-67^+^ and GzB^+^ MAIT cell proportions with one year outcome for LN patients. While ROC analysis of MAIT cell frequency did not reach significance, Ki-67 expression displayed a trend toward significance (**Figure S10**). Remarkably, ROC analysis of GzB production defined a predictive model for complete remission (**Figure 8D**).

## 4 Discussion

In this study, we evaluated the frequency, phenotype and function of peripheral MAIT cells in patients with active or non-active LN. MAIT cell frequency was found to be marked reduced among patients in comparison to HC, consistently with previous reports (27, 28). We furthermore observed that remaining circulating MAIT cells in LN patients displayed a modified phenotype with an increased proportion of CD127^-^ CCR6^-^ cells. One can hypothesized that depletion of peripheral blood MAIT cells during LN development is partly caused by modified expression of CCR6. Indeed, CCR6 is implicated in lymphocyte recruitment to inflamed tissues, and may be involved in T cell migration to kidneys during renal disease development (31, 32). In patients with LN, we hypothesize that MAIT cells may therefore partially disappear from the circulation due to recruitment to inflamed tissues and secondary lymphoid organs. Lower expression of CCR6 by circulating MAIT cells may reflect their recruitment to inflamed renal tissues during SLE progression as shown for other diseases involving pancreas (20), central nervous system (26), adipose tissue (24), kidney (33), and lung (34). It is noteworthy that MAIT cells (CD3^+^Vα7.2^+^IL-18Rα^+^) identified in kidney tissues were mainly located within the glomeruli and tubulointerstitium of patients with class III and IV LN (33). Interestingly, the latter study has demonstrated that suppression of MAIT cell activation impaired autoantibody production and LN development in an animal model. Moreover, tissue infiltration by MAIT cells was also observed in other autoimmune diseases such as arthritis and diabetes (21). Until recently, MAIT cells detection in human tissues, including the kidney, was done by cell–specific TCR transcripts (35, 36). In their recent study, Law et al. characterized by flow cytometry MAIT cell phenotypes from renal-tissue samples obtained from patients with chronic kidney diseases displaying renal fibrosis and from healthy controls (37). In both type of samples, kidney-derived MAIT cells displayed a tissue-resident phenotype, characterized especially by CD103 and CD69 expression. However, CD69 expression on MAIT cells was significantly increased on fibrotic as compared to nonfibrotic kidneys, suggesting that local environment within fibrotic kidneys drives MAIT cells toward a more activated state.

Another explanation for the decrease of circulating MAIT cell frequencies in LN patients could be an increased cell death induced by their activation (28). Indeed, there is a positive correlation between the SLEDAI score and CD69 and CD25 acute activation markers in LN patients. However, this should be confirmed by analyzing other chronic activation markers such as CD38 and HLA-DR. Cytokine-driven activation can induce cell death, which may be in part responsible for MAIT cell depletion. High levels of IL-12 and IL-18 leave MAIT cell vulnerable to persistent activation (28, 38), a mechanism that has also been proposed for invariant NKT cells, another subset of innate-like T cells (39).

A decrease of peripheral MAIT cells has also been described during HIV infection (19, 40, 41). MAIT cells recruitment to tissues has been proposed in response to microbial translocation following the loss of intestinal epithelial barrier integrity during this viral infection. Interestingly, intestinal microbial translocation occurs in both SLE and LN (42, 43). As MAIT cells recognize bacteria-derived ligands, this translocation may induce MAIT cell activation. This hypothesis should be investigated in future studies.

In adults, circulating MAIT cells have display an effector memory phenotype, characterized by expression of chemokine receptors CCR5 and CCR6, as well as high level of CD127. Our data show an increase of CCR6^-^ CD127^-^ double negative cells among remaining circulating MAIT cells in SLE patients with LN, which is the first report in the literature to our knowledge. As hypothesized above, it may be due to migration of double positive CCR6^+^ CD127^+^ cells toward inflamed tissue, or *de novo* differentiation toward terminally differentiated phenotype. CD127 forms the α-subunit of IL-7 receptor (IL-7Rα) that is associated to common γ-chain (CD132) to transmit its signal. IL-7 is the key cytokine for T-cell growth and development, as well as for regulation of naive and memory T-cell related homeostasis or long-term survival. IL-7Rα is indeed expressed by all naive T cells, as well as on memory T cells. On the other side, most CD8^+^ effector T cells and FOXP3^+^ regulatory T cells do not express IL-7α (44). In our study, fewer MAIT cells from class III-IV LN patients express CD127 compared with healthy individuals. Interestingly, loss of CD127 is also observed among CD8^+^ T cells although not significant (not shown). Such phenomenon has been described in CD8^+^ T cells from HIV-infected patients; it has been linked to the shedding or alternative splicing of CD127 induced by IL-7 and mediated by STAT5 and MMP-9, and could be implicated in dysfunctional states of CD8^+^ T cells that remain in the circulation after induction of efficient antiretroviral therapy (45, 46). Among papers analyzing the phenotype of blood MAIT cells in inflammatory diseases, two studies described an increased CD127 expression in T1D (22) and MS patients (47). This increase has been interpreted as a reflect of higher fluorescence mean intensity, which may suggest an increase in the number of molecules at cell surface per MAIT cell, but not an increase in the frequency of CD127^+^ MAIT cells. We and others reported previously rather a decrease of CD127-expressing circulating MAIT cells during severe SARS-CoV-2 infection (34), or chronic hepatitis delta virus infection (38). Contrary to the strong activation phenotype observed in these two papers, LN MAIT cells do not display higher expression of acute activation markers such as CD69 and CD25 in comparison to controls. Moreover, CD127 expression has been shown to be lost by effector T cells (48). Thus, we speculate that a subset of MAIT cells (CCR6^-^ CD127^-^ double negative cells) could be a result from reverting mechanism from effector memory to terminally differentiated memory T cells (TDEM) in the context of proliferative LN. Expansion of TDEM CD8^+^ T cells (CD45RA^+^CCR7^-^CD27^-^CD28^-^) producing high levels of proinflammatory cytokines and cytotoxic activity have been indeed reported in SLE with high disease activity and damage indexes (49) and have been shown to be associated with a higher risk of long-term kidney graft dysfunction (50, 51). Here, contrary to what has been noted in other studies, we do not report an increase in CD27^-^ MAIT cells (**Figure S6**). In HIV patients (41) and patients with type 1 diabetes (52) the CD27^−^ subset has been proposed to potentially represent a terminally differentiated or exhausted MAIT cell subset.

In healthy adults, most MAIT cells are noncycling cells with less than 1% Ki-67^+^ (15). Instead, contrary to controls, in LN patients MAIT cells have a higher proliferating capacity (as defined by Ki-67 expression) in both class III-IV and class V. The systemic loss of MAIT cells may be attributable to increased turnover in LN patients. MAIT cells in LN patients display a decreased expression of KLRG1. KLRG1 is an inhibitory receptor for NK cells, and its expression in T cells is associated with impaired ability to proliferate. In our study, however there was no difference in PD1 expression on MAIT cells from LN patients as compared to controls (see **Figure S6**). This data contrasts with previous observations (19) that could be due to different patient characteristics (LN versus SLE).

In this study, we did not analyze cytokines and GzB production after *in vitro* TCR specific stimulation, since MAIT cells are usually exhausted in chronic inflammatory diseases (20, 24) and respond poorly to such activation. In contrast, restimulation with PMA/ionomycin allow stronger detection of effector molecules and showed increased IL-2, IL-4 and IL-17 expression arguing in favor of a proinflammatory role of MAIT cells in LN notably in classes III-IV. Moreover, increased GzB production detected in CD56^low^ MAIT cells from classes III/IV LN patients suggests their deleterious cytotoxic effect on SLE renal tissues. Multivariable analysis of cytokines and GzB showed that monofunctional MAIT cells producing GzB were more frequent in classes II and III/IV than in class V (see **Figure 7B**). The latter is a less inflammatory class of LN caused by glomerular membranous subepithelial deposition of immune complexes affecting podocytes without significant leukocyte infiltration in the renal tissue. Together, our findings show that MAIT cells in LN display activated and dysfunctional phenotype, which can become predictive factors for the achievement of 1 year remission. In the present study, patients were analyzed once at inclusion and it would be important to perform longitudinal studies after induction therapy in larger cohorts.

In conclusion, the current study described MAIT cells as a new prognostic factor for remission, complications, and relapse rate for LN patient care following induction therapy. High frequency of circulating MAIT cells may represent a favorable prognostic factor for complete remission, whereas high production of GzB is a risk factor for partial or unfavorable response to treatment in lupus nephritis.

## Data availability statement

The raw data supporting the conclusions of this article will be made available by the authors, without undue reservation.

## Ethics statement

The study was approved by the “comité de protection des personnes” (Paris, France) under reference ID-RCB-2014-A00809-38. All patients provided a written informed consent. The studies were conducted in accordance with the local legislation and institutional requirements. The participants provided their written informed consent to participate in this study.

## Author contributions

EL, CB, JDS, LN, LB and NB performed contributions to the acquisition and analysis of data; MP and ED have done all patient consents, ethical approval and clinical analysis; EL, AL, RCM and HF wrote the manuscript; AL, RCM and HF contributed to the conception and design of the work or interpretation of data for the work, revise the manuscript critically for important intellectual content and the final approval of the version to be published; ED, RCM and HF provided agreement to be accountable for all aspects of the work in ensuring that questions related to the accuracy or integrity of any part of the work are appropriately investigated and resolved.
