# Supplementary material for: MAIT cells altered phenotype and cytotoxicity in lupus patients are linked to renal disease severity and outcome
Source: Front Immunol. 2023 Oct 10;14:1205405. doi: 10.3389/fimmu.2023.1205405 (PMC10598677; doi:10.3389/fimmu.2023.1205405)

Antibody (clone)	Compagny	Reference
BV 785 mouse anti-human CD3 (OKT3)	BioLegend	317330
BV 711 mouse anti-human CD4 (OKT4)	BioLegend	317440
PE Cy5 mouse anti-human CD4 (OKT4)	BioLegend	317412
APC Cy7 mouse anti-human CD8 (SK1)	BDbiosciences	557834
BV 421 mouse anti-human Vα7.2 (3C10)	BioLegend	351716
PE Cy7 mouse anti-human CD25 (M-A251)	BDbiosciences	557741
FITC mouse anti-human PD1 (MIH4)	BDbiosciences	557861
PE CF594 mouse anti-human TCRγδ (B1)	BDbiosciences	562511
BV 605 mouse anti-human CD161 (HP-3G10)	BioLegend	339916
PE mouse anti-human CCR6 (G034E3)	BioLegend	353410
PE Cy5 mouse anti-human CD127 (R34.34)	Beckman Coulter	A64617
BV 510 mouse anti-human CD56 (HCD56)	BioLegend	318340
BV 650 mouse anti-human CD69 (FN50)	BioLegend	310934
PE mouse anti-Ki67 Set (B56)	BDbiosciences	556027
APC mouse anti-human BTLA (MIH26)	BioLegend	344510
BV510 mouse anti-human CD27 (O323)	BioLegend	302836
PE-Cy7 hamster anti-mouse/humanKLRG1 (2F1)	BioLegend	138416
PE rat anti-human IL2 (MQI-17H12)	BioLegend	500307
PE Cy7 mouse anti-human IL4 (8D4-8)	BDbiosciences	560672
APC rat anti-human IL10 (JES3-19F1)	BDbiosciences	562036
BV 711 mouse anti-human IL17a (BL168)	BioLegend	512328
BV 510 mouse anti-human Granzyme B (GB11)	BDbiosciences	563388
BV 650 mouse anti-human TNFα (Mab11)	BioLegend	502938
FITC mouse anti-human IFNγ (4S.B3)	BioLegend	502507

Figure S1
Monoclonal antibodies used in the study.

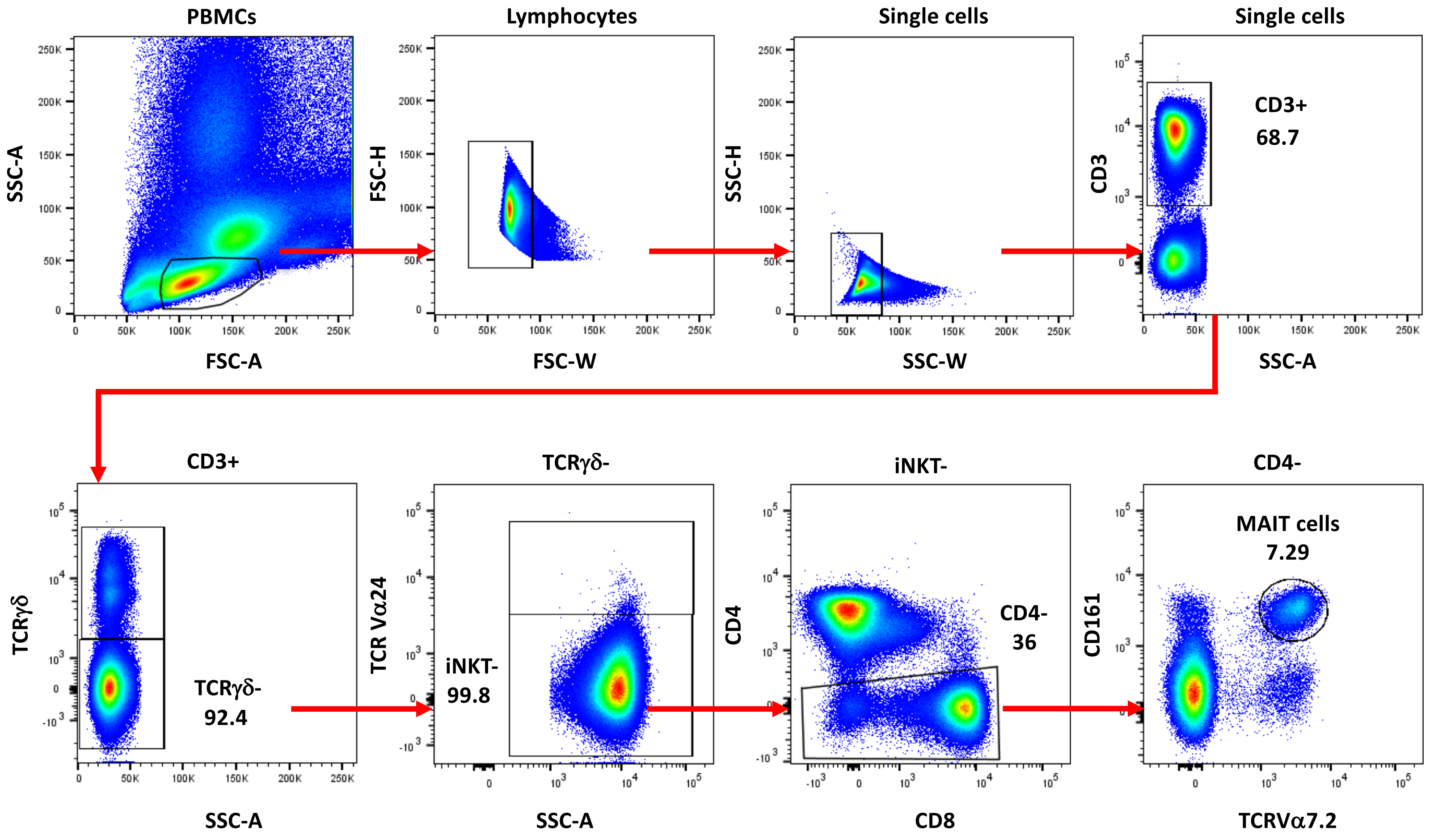

Figure S2. Representative gating strategy of peripheral MAIT cells.

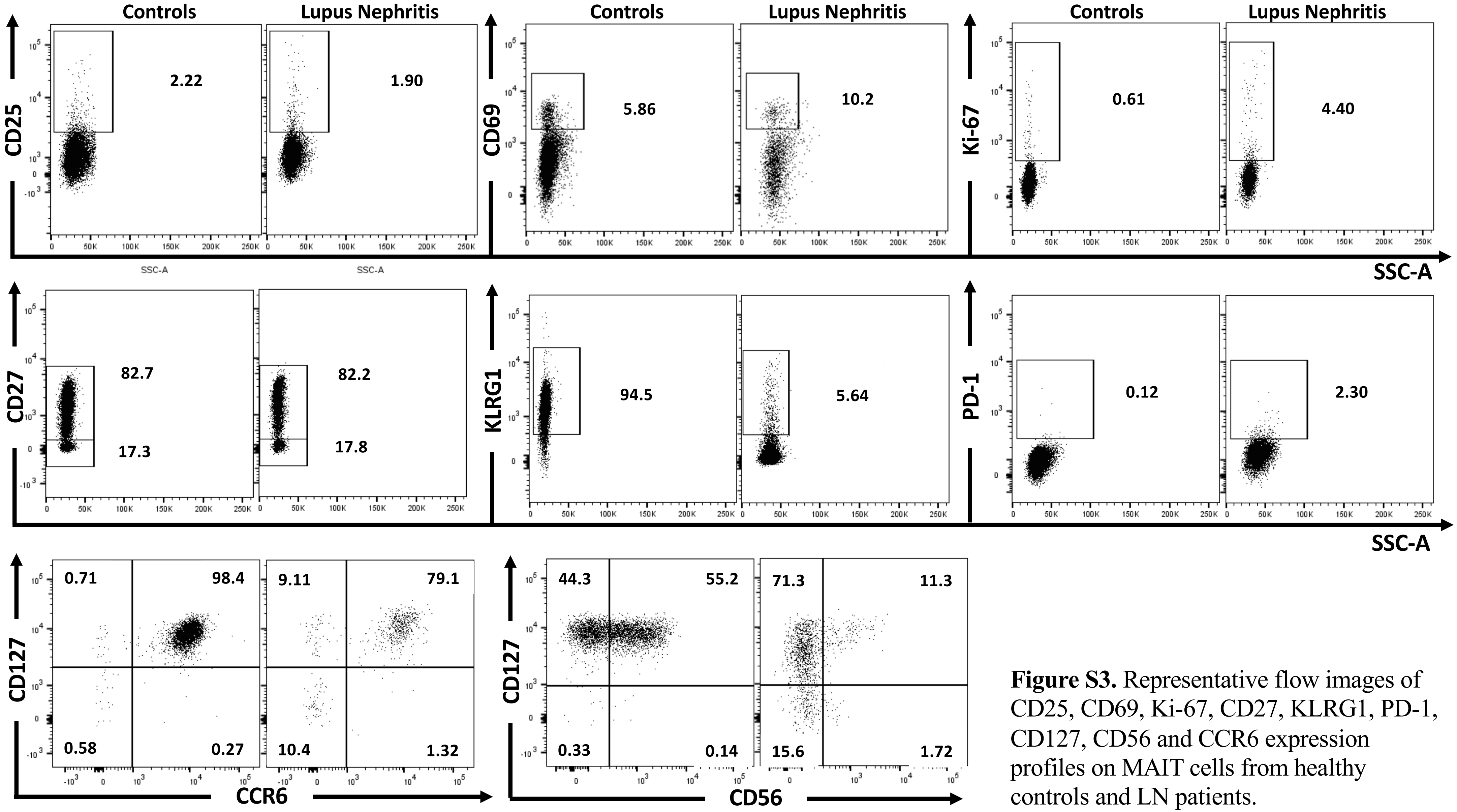

Figure S3. Representative flow images of CD25, CD69, Ki-67, CD27, KLRG1, PD-1, CD127, CD56 and CCR6 expression profiles on MAIT cells from healthy controls and LN patients.

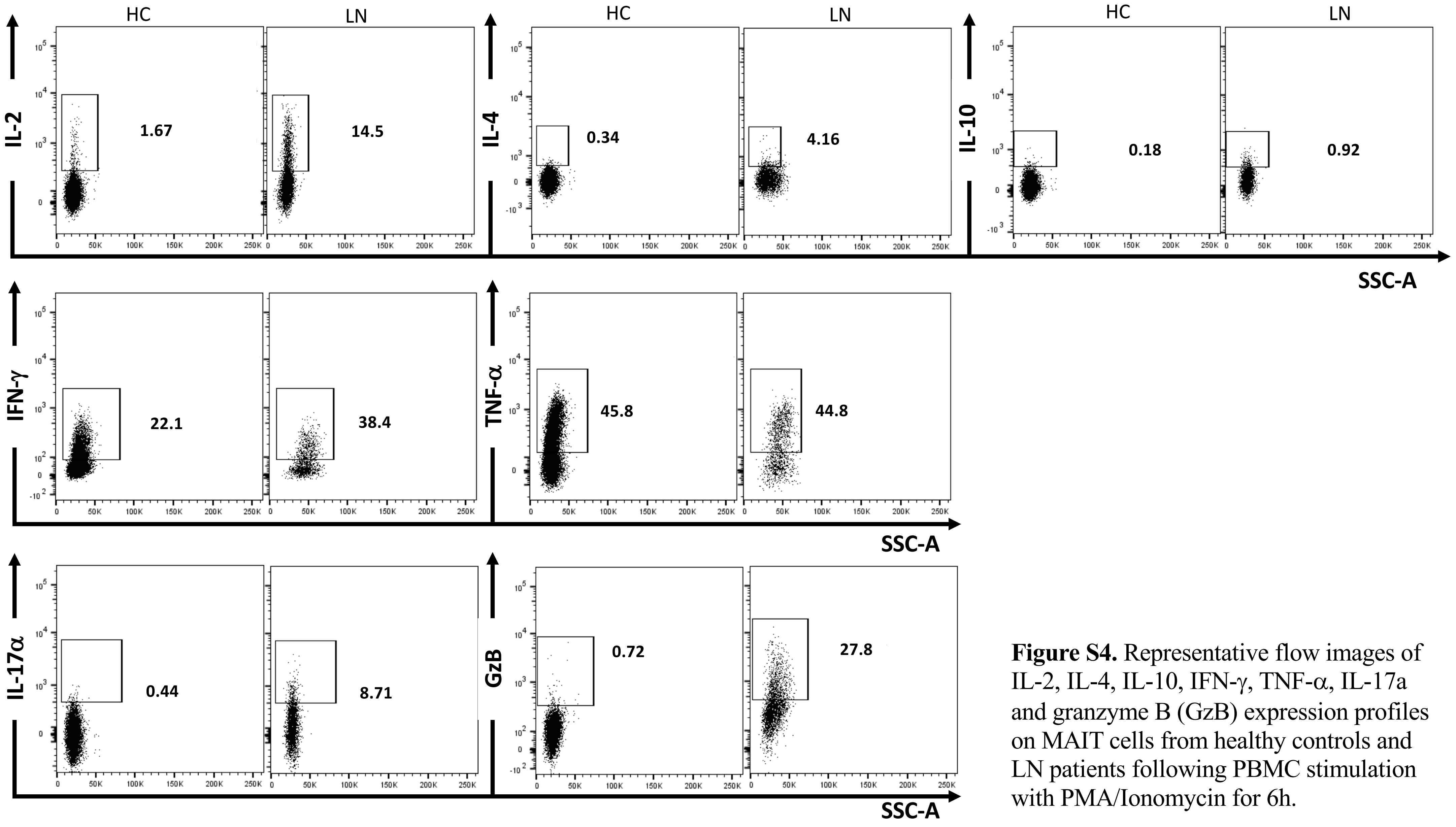

Figure S4. Representative flow images of IL-2, IL-4, IL-10, IFN-γ, TNF-α, IL-17a and granzyme B (GzB) expression profiles on MAIT cells from healthy controls and LN patients following PBMC stimulation with PMA/Ionomycin for 6h.

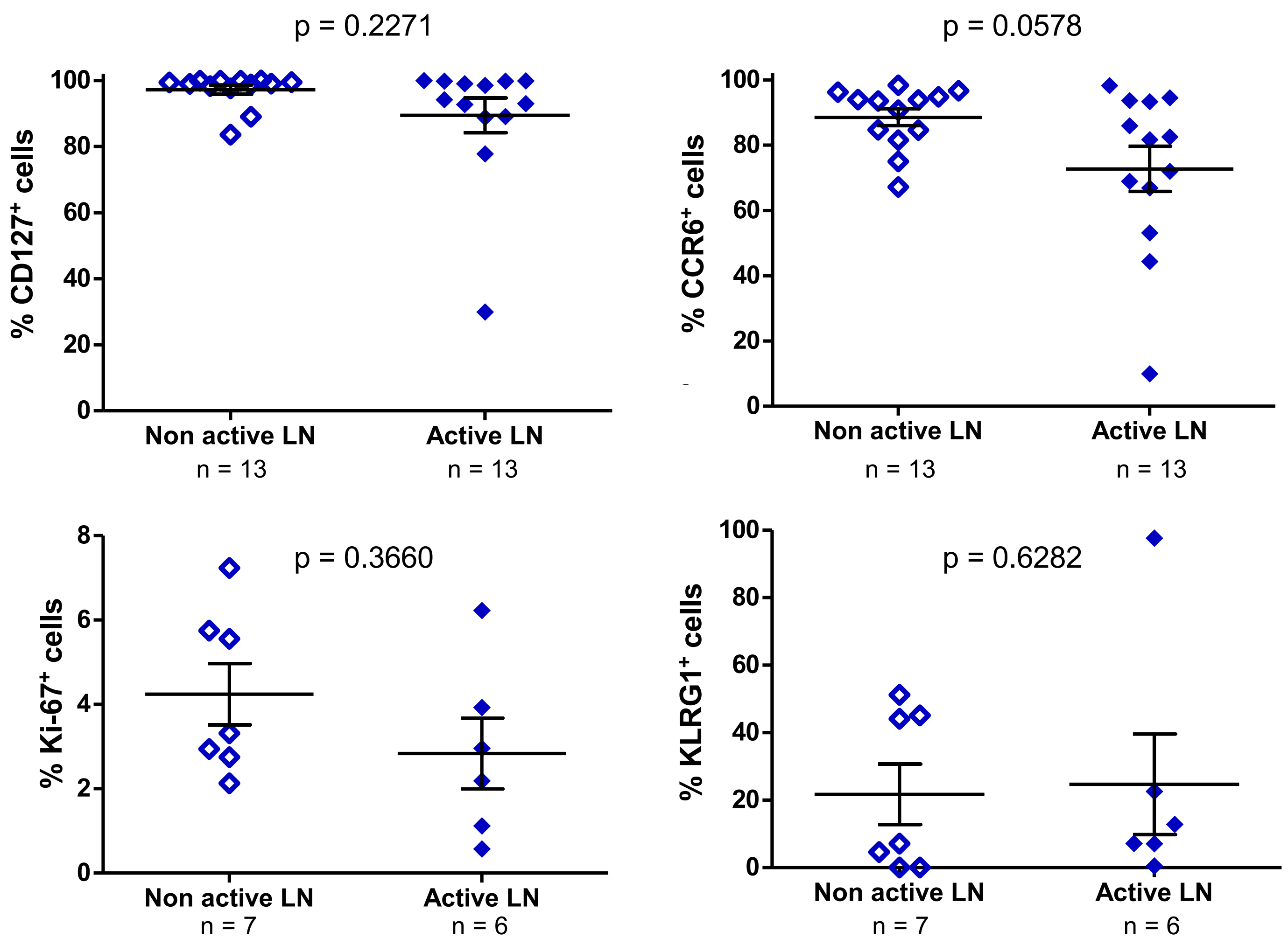

Figure S5. Percentage of MAIT cells expressing CD127, CCR6, Ki-67 and KLRG1 in patients with active (III or IV +/-V) or non active (II and pure class V) lupus nephritis. Data was analysed with Mann-Whitney U-tests. Horizontal lines are mean ± SD values. The number of subjects are indicated below each dot plot. A single experiment was performed for each individual control or patient.

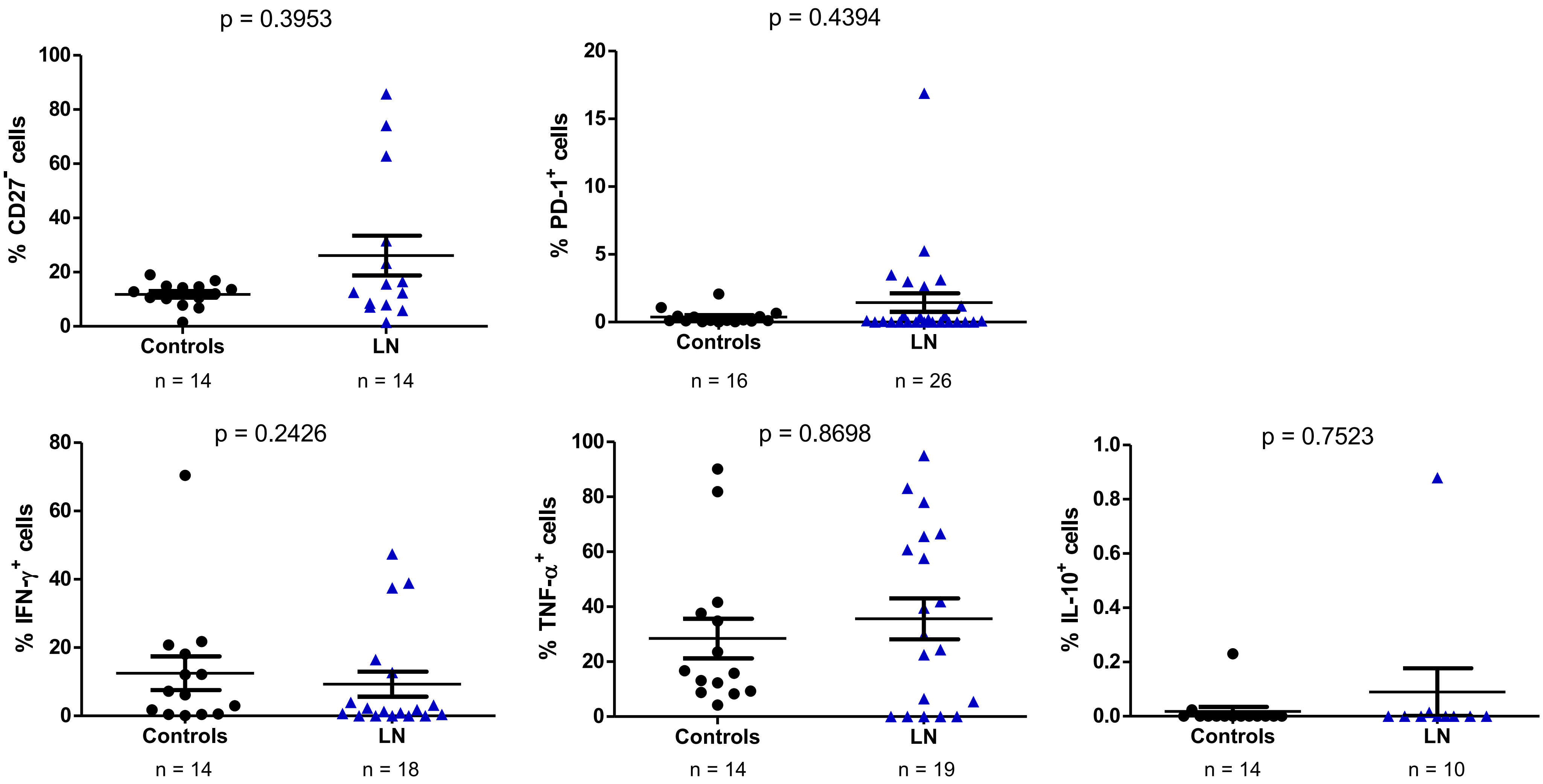

Figure S6. Percentage of $CD27^-$, PD-1+, IFN-γ^+, TNF-α^+ and IL-10^+ MAIT cells in controls and patients. Data was analysed with Mann-Whitney U-tests. Horizontal lines are mean ± SD values. The number of subjects are indicated below each dot plot. A single experiment was performed for each individual control or patient.

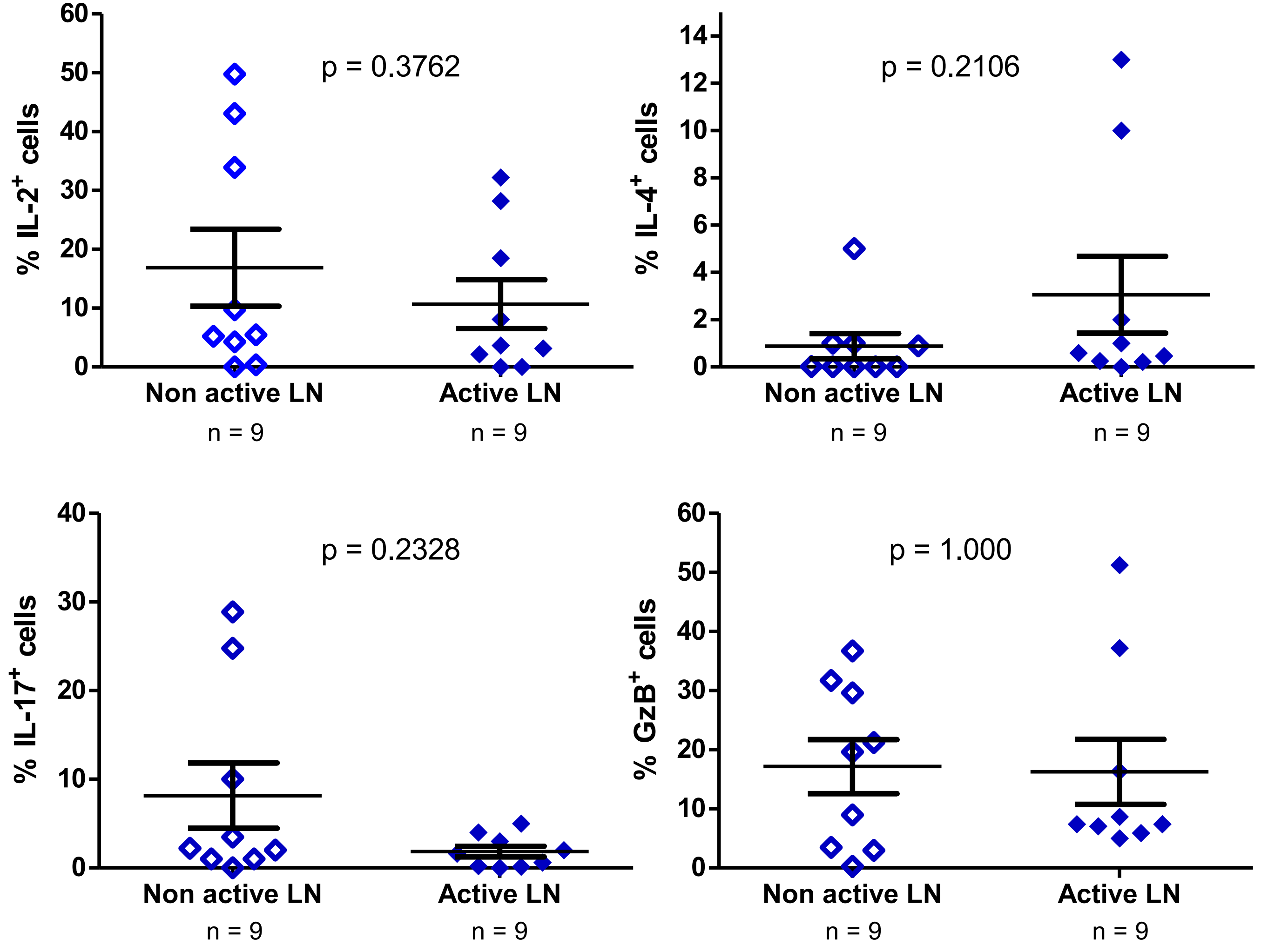

Figure S7. Percentage of MAIT cells expressing IL-2, GzB, IL-4 and IL-17 in patients in patients with active (III or IV +/-V) or non active (II and pure class V) lupus nephritis. Data was analysed with Mann-Whitney U-tests. Horizontal lines are mean ± SD values. The number of subjects are indicated below each dot plot. A single experiment was performed for each individual control or patient.

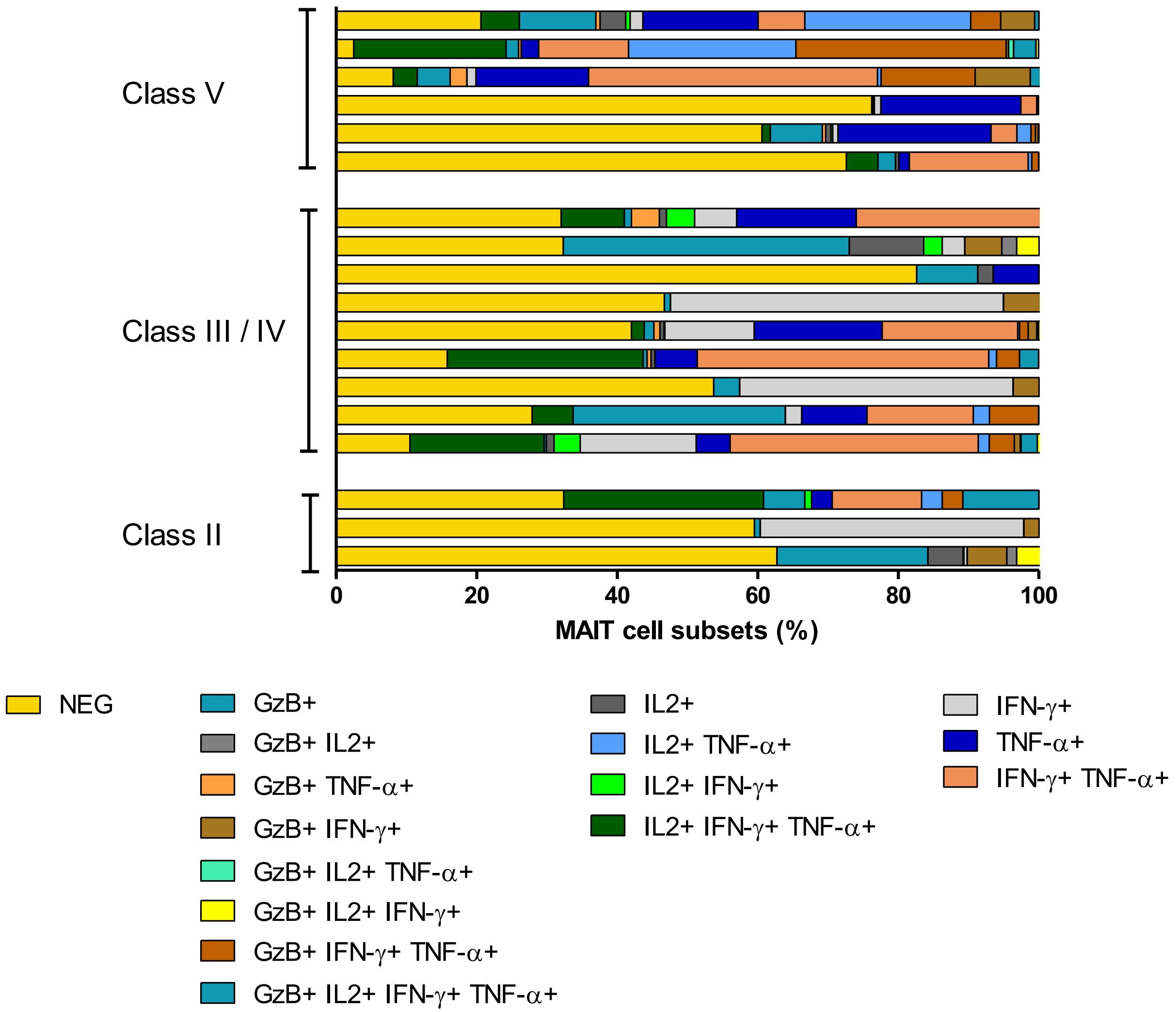

Figure S8. Individual profiles of cytokine production by MAIT cells, including IL-2, TNF-α, IFN-γ and/or GzB, among LN patients of class II (n=3), III / IV (n=9) and V (n=6). A single experiment was performed for each individual control or patient.

Figure S9. Correlation between MAIT cell frequency, GzB frequency, GzB production, and Ki-67 expression with clinical data. UPCR, urine protein/ creatinine ratio; eGFR, estimated glomerular filtration rate. The number of subjects are indicated for each graph. A single experiment was performed for each individual control or patient.

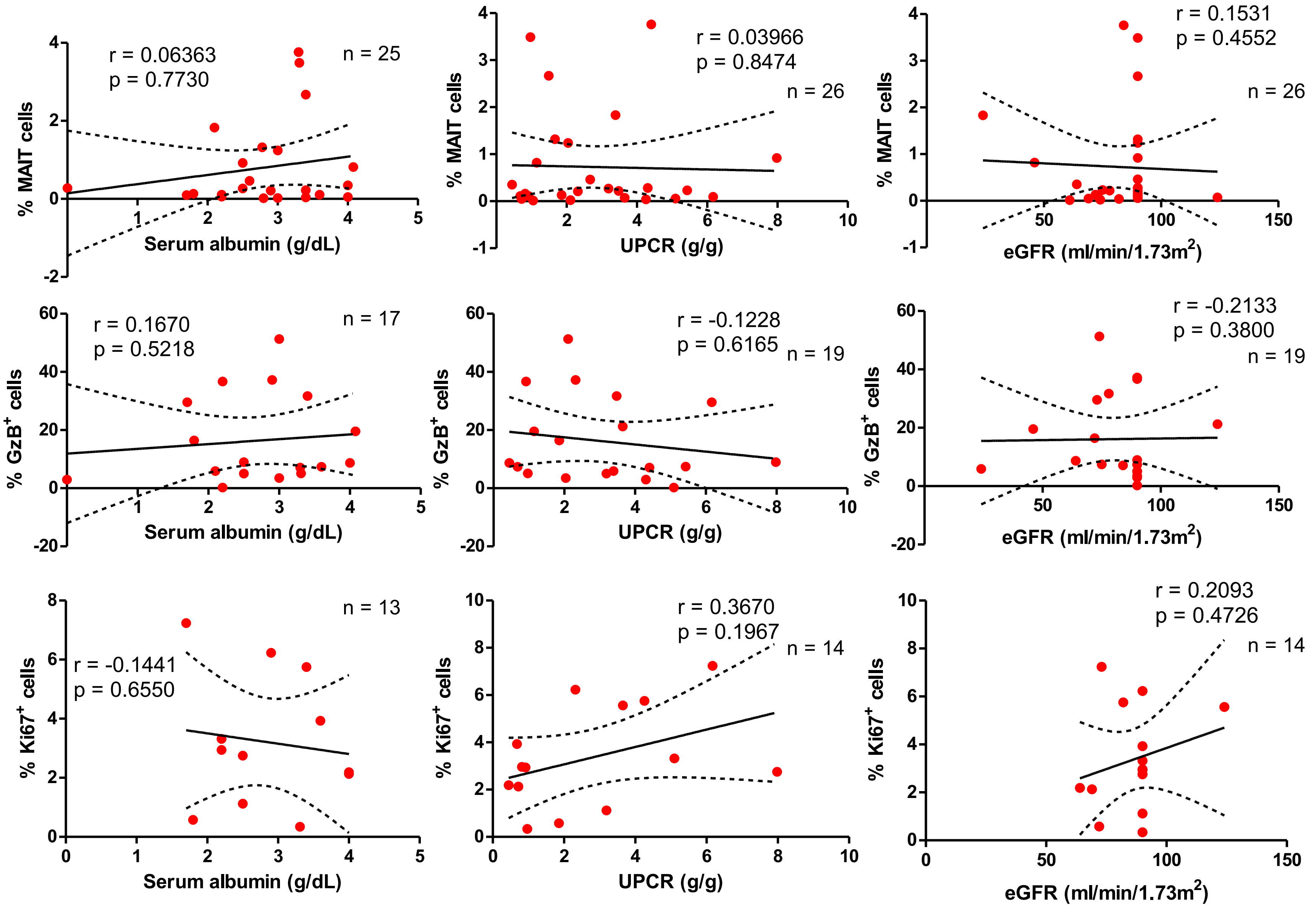

Figure S10. Receiver operating characteristic (ROC) curve of MAIT cell frequency or Ki-67 expression and sensitivity for remission prediction at one year outcome after induction therapy.

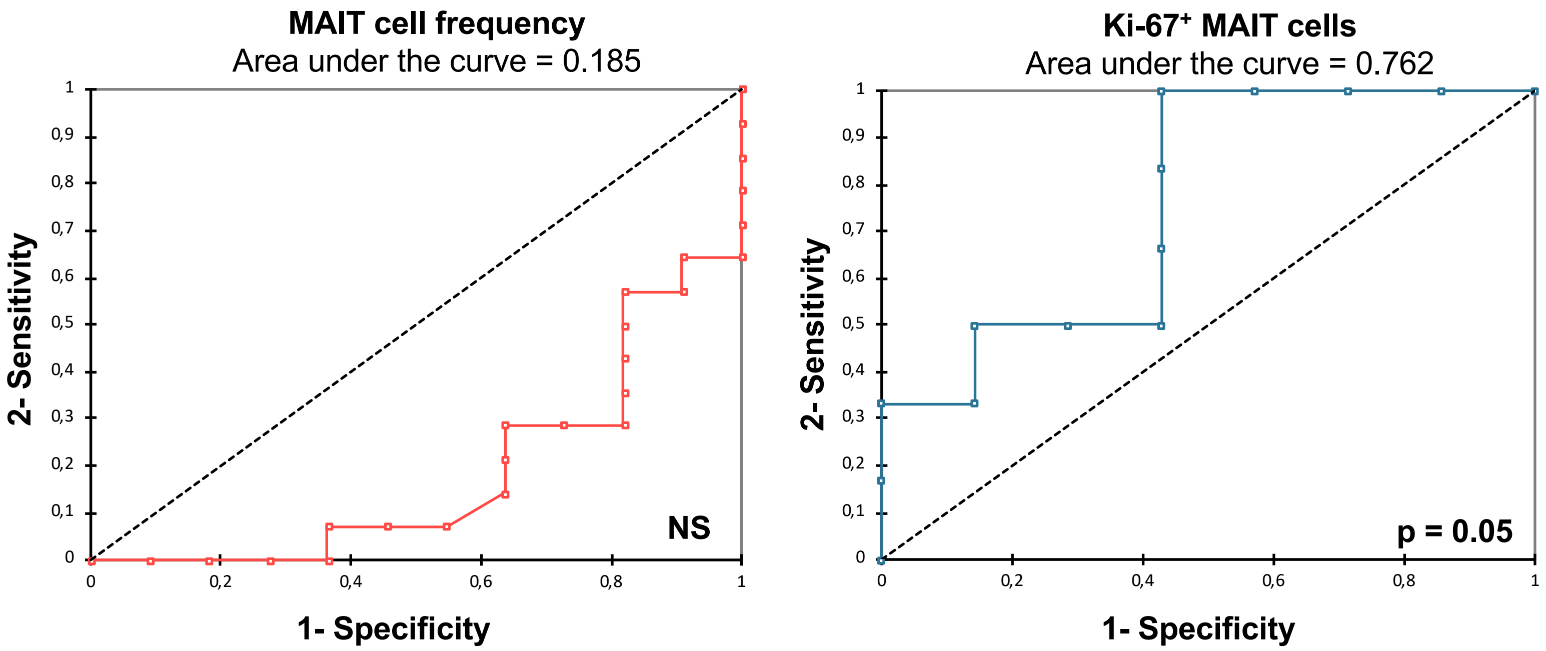

Supplement: Supplementary file 1 [file Presentation_1.pdf]
